# Similar Trends in Serum VEGF-D Levels and Kidney Angiomyolipoma Responses with Longer Duration Sirolimus Treatment in Adults with Tuberous Sclerosis

**DOI:** 10.1371/journal.pone.0056199

**Published:** 2013-02-20

**Authors:** Izabela A. Malinowska, Nancy Lee, Vidhya Kumar, Elizabeth A. Thiele, David Neal Franz, Stephen Ashwal, Arthur Sagalowsky, Francis J. DiMario, Drew Cutler, Darcy Krueger, Susana Camposano, Jan Paolini, Sandra L. Dabora

**Affiliations:** 1 Department of Medicine, Brigham and Women’s Hospital, Boston, Massachusetts, United States of America; 2 Department of Neurology, Massachusetts General Hospital, Boston, Massachusetts, United States of America; 3 Department of Pediatrics, Cincinnati,Children’s Medical Center, Cincinnati, Ohio, United States of America; 4 Department of Neurology, Loma Linda University, Loma Linda, California, United States of America; 5 Department of Urology, University of Texas Southwestern, Dallas, Texas, United States of America; 6 Department of Pediatric Neurology, Connecticut Children’s Medical Center, Hartford, Connecticut, United States of America; 7 Pediatric Nephrology, Loma Linda University, Loma Linda, California, United States of America; University of Washington, United States of America

## Abstract

**Context:**

We have previously shown that serum VEGF-D is elevated at baseline, correlates with kidney angiomyolipoma size at baseline and 12 months, and decreases with sirolimus treatment in adults with tuberous sclerosis complex (TSC). To further investigate the utility of serum VEGF-D for longer term monitoring of TSC kidney disease, we present VEGF-D level results with 24 month follow-up.

**Objective:**

To compare 24 month VEGF-D levels in two subgroups of sirolimus treated patients (OFF SIROLIMUS AFTER 12 MONTHS or ON SIROLIMUS AFTER 12 MONTHS).

**Design and Intervention(s):**

Serum VEGF-D was measured in samples collected from subjects enrolled in a phase 2 multicenter trial evaluating sirolimus for the treatment of kidney angiomyolipomas associated with TSC or TSC/LAM. All participants were treated with sirolimus from 0–12 months. During months 12–24, sirolimus was discontinued in one subgroup. The other subgroup was treated with additional sirolimus.

**Setting:**

Adult TSC participants were recruited from six clinical sites in the United States (comprehensive TSC clinics, 5; urology clinic, 1).

**Patients:**

There were 28 TSC patients who completed all 24 months of the study and serum samples were available at 24 months from 18/28 patients.

**Main Outcome Measure(s):**

We compared the percent change in VEGF-D levels (baseline to 24 months) in patients from the two treatment subgroups.

**Results:**

At 24 months, VEGF-D levels decreased by 67% compared with baseline (to 787±426 pg/ml) in the ON SIROLIMUS AFTER 12 MONTHS group versus a 13% decrease (to 2971±4014 pg/ml) in the OFF SIROLIMUS AFTER 12 MONTHS group (p = 0.013, Mann-Whitney test). A similar trend was observed in kidney angiomyolipoma size but not in pulmonary function tests.

*Conclusions* Serum VEGF-D may be useful for monitoring response to treatment with sirolimus and kidney angiomyolipoma size in patients with TSC, but confirmation is needed.

**Trial Registration:**

Clinical trials.gov NCT00126672.

## Introduction

Vascular endothelial growth factors (VEGFs) are molecules that stimulate the development of vessels during embryogenesis and growth of new vessels in mature organisms after tissue injury, inflammation, infarct/ischemia, or during neoplastic vascularisation. There are several VEGF isoforms and in humans these include VEGF A–D. VEGF-D binds to VEGF receptors 2 and 3 (VEGFR2, VEGFR3), localized on endothelial cell membranes. VEGF-D plays an important role in lymphatic vessel development and regrowth [1].

Several groups have shown that serum VEGF-D levels are elevated in cohorts of sporadic pulmonary lymphangioleiomyomatosis (LAM) patients [2], [3], [4]. Sporadic LAM is an uncommon interstitial pulmonary disorder that can cause end stage lung disease in women [5]. Although questions remain about the mechanism that leads to high serum VEGF-D levels, these observations suggest it could be a useful diagnostic biomarker for LAM. Because VEGF-D testing may be a non-invasive alternative to lung biopsy for diagnosing LAM in women with cystic lung disease of unknown etiology [6], serum VEGF-D testing is now available for clinical use (see http://www.thelamfoundation.org/, VEGF-D TEST, VEGF-D quantification sample submission form).

Tuberous sclerosis complex (TSC) is a genetic disorder that shares important molecular pathology and clinical features with sporadic LAM. These features include: 1) *TSC1/TSC2* gene mutations have been identified in lung and other tissues from sporadic LAM patients [7], [8], [9], [10], [11]; 2) activation of the mTOR (mammalian Target Of Rapamycin) pathway occurs in abnormal LAM and TSC tissues [12], [13]; 3) LAM is a major feature of TSC [14] and cystic lung disease consistent with early LAM is present in 30–40% of women with TSC [15], [16]; 4) kidney angiomyolipomas are a major feature of TSC and occur in 40–50% of individuals with sporadic LAM [5], [17].

Based on the understanding that constitutively activated mTOR signaling is a pathologic feature of TSC and sporadic LAM, there has been recent progress in clinical trials evaluating mTOR inhibitors for the treatment of TSC and/or LAM. Clinical trial results show that mTOR inhibitor treatment results in tumor regression and improved lung function in patients with TSC and/or LAM. Kidney angiomyolipoma regression was observed in patients with TSC and/or LAM treated with sirolimus in three phase 2 studies [18], [19], [20]. TSC related brain tumors (subependymal giant cell astrocytomas, also known as SEGAs) decreased in size when treated with either sirolimus [19] or the rapamycin analog, everolimus [21]. In a phase 3 trial, improved lung function was observed with sirolimus treatment in patients with LAM [22].

The potential utility of serum VEGF-D as a biomarker for TSC and/or LAM was evaluated in two sirolimus trials for TSC and/or LAM patients. McCormack and co-workers (2011) observed that VEGF-D levels were high at baseline and decreased with sirolimus treatment (at 6 and 12 months), but these investigators did not correlate VEGF-D levels with lung function or other parameters. In our multicenter kidney angiomyolipoma study, we observed that VEGF-D levels were high at baseline, decreased with sirolimus treatment during the first year, and that VEGF-D levels correlated with kidney angiomyolipoma size but not lung function [19]. Here we report the 24 month VEGF-D data for two treatment subsets of patients from our phase 2 multicenter trial: ON or OFF SIROLIMUS AFTER 12 MONTHS.

## Methods

The CONSORT checklist and protocol (original and final versions) have been published previously in PLoS One (see supporting information files Protocol S1, Protocol S2, and Checklist S1 in reference [19]). A detailed description of protocol deviations, violations, and amendments has been reported previously (see methods section and supporting information file Table S13 in reference [19]).

### Patients, Clinical Study, and Serum Samples

**Figure 1:**
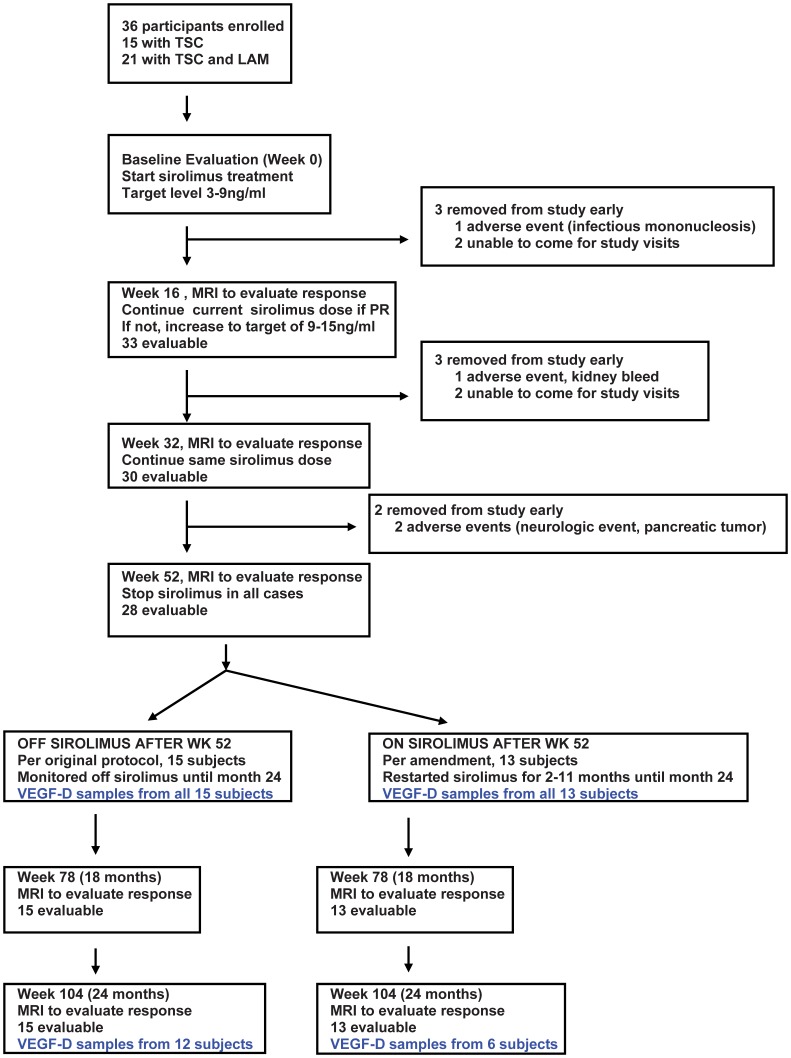
Enrollment and VEGF-D sample chart.

Serum samples were collected from patients enrolled in a multicenter, phase 2 trial evaluating the safety and efficacy of sirolimus for the treatment of kidney angiomyolipomas associated with TSC [19]. This protocol was approved by the institutional review boards (IRBs) at all participating institutions (Dana-Farber Cancer Institute IRB, University of Texas Southwestern Medical Center IRB, Connecticut Children’s Medical Center IRB, Cincinnati Children’s Medical Center IRB, NYU School of Medicine IRB, Loma Linda University IRB). Written informed consent was obtained from all participants. Of 36 enrolled subjects, 28 completed all required clinical imaging and laboratory testing during the 2 year study period. Sufficient serum samples from all time points (baseline, 4, 8, 12, and 24 months) were available from 18 subjects for the VEGF-D analysis (Figure 1). All subjects were treated with sirolimus during the first year. During months 12–24, there was one subgroup that received no additional sirolimus (n = 15), and one subgroup that was treated with additional sirolimus (n = 13). Treatment assignment was not random; this additional sirolimus treatment was offered per a protocol amendment to those individuals who had a partial response or stable disease if the treating study physician felt it was in the best interest of the participant. There were 14 patients who were eligible for sirolimus treatment during year 2 because the others had completed (or almost completed) all 24 months by the time the amendment was approved (see additional details in reference 19. Because a comparison of VEGF-D levels from two groups with different treatments from months 12–24 was not part of the original study design, the comparison reported here is an exploratory post-hoc analysis. Samples of serum VEGF-D testing were available from 12 subjects from the OFF SIROLIMUS AFTER 12 MONTHS group and 6 subjects from the ON SIROLIMUS AFTER 12 MONTHS group (Figure 1). These two subgroups had similar tumor sizes and VEGF-D levels at baseline and 12 months (see Tables S1 and S2).

The percent change in tumor size was determined using magnetic resonance imaging (MRI) before and after treatment according to the Response Evaluation Criteria in Solid Tumors (RECIST) [23] as previously described [19]. We have previously used Spearman correlation to show a significant correlation between kidney tumor size and VEGF-D levels at baseline and 12 months in this group of patients (see Figure 4 in reference 19). Pulmonary function parameters (FVC, FEV1, and DLCO) were measured in female participants at baseline and 12 months using standard spirometry [19].

Sirolimus treatment was initiated with a loading dose of 6 mg by mouth on day 1 followed by 2 mg by mouth daily. Sirolimus is FDA approved for use after kidney transplantation and this is the standard starting dose used in the kidney transplant population. The dose was then adjusted to maintain a target trough blood level of 3–15 ng/ml. There was a wide range of sirolimus doses required to maintain target trough levels. At 12 months, the mean dose was 6.7 mg per day. The lowest dose was 1 mg every other day and the highest dose was 24 mg per day. Additional details regarding dose adjustments and the wide dose range have been described previously [19].

Complete data on the adverse events that were observed during this 2 year study have been reported previously so these details are not repeated here (see Table 2, Table S2, and Table S12 in reference 19). Summarizing toxicity data for all subjects during the entire enrollment period: drug related mild to moderate (grade 1–2) toxicities that occurred with a frequency of >20% included stomatitis, hypertriglyceridemia, hypercholesterolemia, bone marrow suppression (anemia, mild neutropenia, leucopenia), proteinuria, and joint pain. There were three drug related severe (grade 3) adverse events that occurred during the first 12 months of treatment: lymphopenia, headache, weight gain. Longer duration sirolimus treatment was well tolerated. During months 12–24 there were no drug related grade 2 or 3 adverse events, there were 5 patients with grade 1 events, and there were 23 with no drug related toxicities.

### Serum VEGF-D ELISA Assay

VEGF-D levels were measured using ELISA (Quantikine Human VEGF-D Immunoassay kit from R&D Systems). Serum was collected using standard red top serum vacutainer tubes with no additive. After clot formation and low speed centrifugation, serum was aliquoted, frozen, shipped overnight on dry ice to the Dabora Lab (Brigham and Women’s Hospital, Boston, MA), and then stored at −80°C until all samples were available for VEGF-D analysis. The manufacturer’s instructions were followed with the following modifications: 1) we included concentrations of 125, 250, 500, 1000, 2000, 4000, 8000, 16000, 32000 pg/ml to generate the standard curve; 2) we used a correction wavelength of 550 nm (instead of 540 nm) because 540 nm was not an option on our plate reader (THERMOmax microplate reader with SoftMax Pro v5 software, Molecular Devices Corp.); 3) sample VEGF-D concentrations were extrapolated from the standard curve using Prism software (version 4.01) and the one-site competition option in order to optimize the fit of the standard curve [24]. All standards were run in quadruplicate (all readings were within 25% of the mean of all 4 readings). All serum samples were run in duplicate and duplicates deviated by no more than 25% from the average of the two readings. During assay optimization, we did observe some plate to plate variability in VEGF-D results from identical samples. For all data reported in this study, we ran all time points for individual subjects on the same plate so VEGF-D concentrations for all time points for individual subjects were extrapolated from the same standard curve.

### Statistical Analysis

GraphPad Prism software (version 4.01) was used for all data analysis, with a p-value ≤0.05 suggesting statistical significance in this post-hoc exploratory analysis. The Mann-Whitney test and paired t test were used to compare VEGF-D levels at baseline and 12 months in patient subgroups. Spearman correlation was used to investigate the correlation between VEGF-D levels and pulmonary function parameters (FVC, FEV1, DLCO and FEV1/FVC).

## Results

### Comparison of 24 Month VEGF-D Levels and Kidney Tumor Regression in 2 Treatment Subgroups

**Figure 2:**
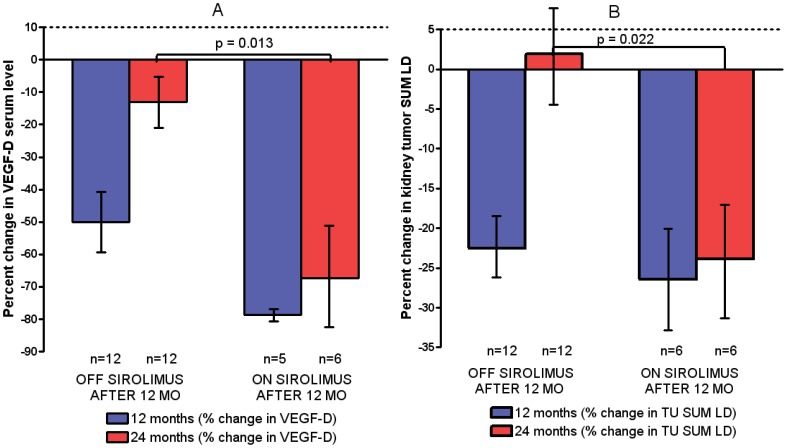
Percent change in serum VEGF-D levels and kidney angiomyolipoma size with sirolimus treatment at 12 and 24 months. A) The blue bars show the mean percent change in serum VEGF-D levels with sirolimus treatment at 12 months for two treatment subgroups. The red bars show 24 month data. Error bars indicate standard error; p = 0.013 at 24 months and NS (p = 0.065) at 12 months. B) Changes in kidney angiomyolipoma size showed a similar trend. The blue bars show the mean percent change in kidney angiomyolipoma size at 12 months for two treatment subgroups. The red bars show the data for year 2 (24 months). Error bars indicate standard error; p = 0.022 at 24 months and NS (p = 0.54) at 12 months.

VEGF-D levels at all time points for both treatment groups are shown in Table 1. As shown in Figure 2A, at 12 months there was a similar percent decrease (compared with baseline) in serum VEGF-D levels in the OFF SIROLIMUS AFTER 12 MONTHS group (50%) and the ON SIROLIMUS AFTER 12 MONTHS group (79%) as expected because there were no treatment differences during the first 12 months. However at 24 months, VEGF-D levels returned toward baseline in the OFF SIROLIMUS AFTER 12 MONTHS group (13% decrease compared with baseline), but not in the ON SIROLIMUS AFTER 12 MONTHS group (67% decrease compared with baseline). This difference was significant (p = 0.013, Mann-Whitney test, see Figure 2).

**Table 1:** VEGF-D levels over time from baseline to 24 months.

	Baseline (0 months)	4 months	8 months	12 months	24 months
**OFF SIROLIMUS AFTER 12 MONTHS**					
n	12	6	9	12	12
VEGF-D mean (pg/ml)	3403	877	922	1000	2971
SD	4098	304	856	752	4014
min	560	539	505	438	506
max	13258	1318	3167	2687	13497
lower 95% CI	800	558	263	523	421
upper 95% CI	6008	1198	1581	1479	5522
VEGF-D median (pg/ml)	1506	817	620	678	1351
VEGF-D geometric mean (pg/ml)	1992	834	747	822	1674
**ON SIROLIMUS AFTER 12 MONTHS**					
n	6	6	6	5	6
VEGF-D mean (pg/ml)	4518	1749	1104	1070	787
SD	2407	875	494	397	426
min	652	687	371	660	322
max	6845	3132	1624	1698	1406
lower 95% CI	1992	831	585	576	340
upper 95% CI	7046	2668	1623	1564	1236
VEGF-D median (pg/ml)	5063	1702	1233	1061	833
VEGF-D geometric mean (pg/ml)	3587	1561	983	1015	681

The trend in VEGF-D levels at 24 months was similar to the trend in kidney angiomyolipoma size (Figure 2). On average, kidney angiomyolipoma response was maintained in the ON SIROLIMUS AFTER 12 MONTHS group, but returned almost to baseline in the OFF SIROLIMUS AFTER 12 MONTHS group. As previously reported [19], kidney angiomyolipoma regression was observed with 12 months of sirolimus treatment. The overall response rate was 44.4%; 16/36 had a partial response using RECIST criteria [23] which was defined as ≥30% decrease in the sum of the longest diameters (sum LD). The remaining patients had stable disease (47.2%, 17/36), or were unevaluable because they came off study prior to any follow-up MRI imaging (8.3%, 3/36). The mean decrease in sum LD was 29.9% (n = 28 at 12 months). The percent change in the kidney tumor size (sum LD) at 24 months for the OFF SIROLIMUS AFTER 12 MONTHS group was 1.1% (n = 15), but was −34.0% in the ON SIROLIMUS AFTER 12 MONTHS group (n = 13) and this difference was significant (p = 0.0005, Mann-Whitney test). As shown in Figure 2B, similar findings were observed if patients with insufficient VEGF-D samples were excluded: the percent change in the kidney tumor size (sum LD) at 24 months for the OFF SIROLIMUS AFTER 12 MONTHS group was 1.9% (n = 12), but was −23.8% in the ON SIROLIMUS AFTER 12 MONTHS group (n = 6); this difference was significant (p = 0.022, Mann-Whitney test). On average, tumor regrowth back to baseline was observed in the OFF SIROLIMUS AFTER 12 MONTHS group, but not in the ON SIROLIMUS AFTER 12 MONTHS group (see Figure S1 for details on individual subjects).

### VEGF-D Levels Over Time and in Subgroups

**Figure 3:**
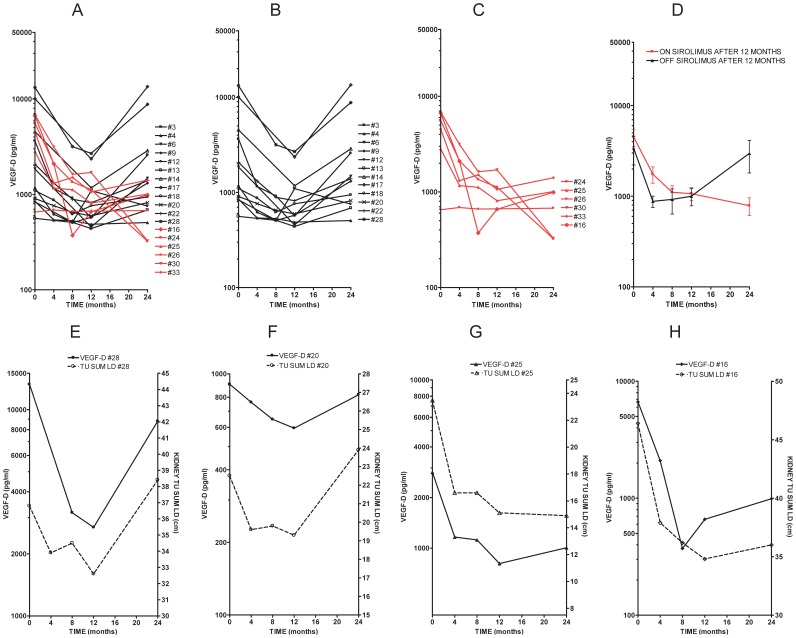
Individual trends in serum VEGF-D levels from subjects with TSC and angiomyolipomas who were treated with sirolimus. VEGF-D levels were measured at baseline before sirolimus was started, and at 4, 8, 12, and 24 months. A) All subjects; B) Subset OFF SIROLIMUS AFTER 12 MONTHS (n = 12, in black); C) Subset ON SIROLIMUS AFTER 12 MONTHS (n = 6, in red); D) Mean VEGF-D levels ± standard error (SEM) for both subsets. Panels E–H) Trends in VEGF-D serum levels and changes in kidney tumor size for individual subjects over time from baseline to 24 months. As indicated, panels E and F show trends for individual subjects (#28-with TSC/LAM and #20-with TSC only) in the OFF SIROLIMUS AFTER 12 MONTHS group; panels G and H show data for the ON SIROLIMUS AFTER 12 MONTHS group (#25-with TSC/LAM and #16-with TSC only). The trends in VEGF-D levels are similar to the trends in kidney angiomyolipoma size in both treatment subgroups.

We compared VEGF-D trends over time from baseline to 24 months in individual subjects. As shown in Figure 3, VEGF-D levels decrease from baseline to 12 months in all subjects during sirolimus treatment. VEGF-D levels then had an upward trend toward baseline levels from months 12–24 in the OFF SIROLIMUS AFTER 12 MONTHS group, but stayed lower than baseline in the ON SIROLIMUS AFTER 12 MONTHS group. See Figure 2, A–D for VEGF-D level trends in individual cases and mean VEGF-D levels for both treatment subgroups. The similarity between the trend in VEGF-D levels and kidney angiomyolipoma size for individual subjects is shown in Figure 3, E–H.

We also evaluated VEGF-D level changes in subgroups at baseline and at 12 months. We noted previously that baseline VEGF-D levels were higher in females compared to males, and in TSC/LAM patients compared to TSC patients. Here we show that the decrease in VEGF-D levels with 12 months of sirolimus treatment occurred in all subgroups, but was most dramatic in females and patients with TSC/LAM (see Figure S2).

We have previously shown a correlation between baseline VEGF-D levels and kidney tumor size. In contrast, here we show the absence of a correlation with baseline pulmonary function tests (FEV1, FVC, DLCO and FEV1/FVC) in this cohort, see Figure S3. We also note that although the mean VEGF-D levels were higher in the subset of subjects with a SEGA (brain tumor associated with TSC; Figure S4), the difference was not significant. However, the power of the PFT and SEGA analyses is limited due to the small sample size.

## Discussion

Our two year follow-up VEGF-D results indicate that VEGF-D level trends may be useful for monitoring kidney angiomyolipoma size following treatment with sirolimus. This study has major limitations due to the small size, non-random assignment of additional sirolimus treatment after month 12, the post-hoc exploratory nature of the study, no corrections for multiple testing, and missing samples (see Figure 1). Although this was a post-hoc analysis, it is important to note that there were no major differences in initial tumor size in those that got more or less drug after 12 months. Furthermore, all patients that completed 12 months on study (n = 28) had stable disease or a partial response at 12 months so all would have been eligible for additional treatment during months 12–24 based on tumor response criteria. Some (14/28) were not eligible because they had completed all or almost all 24 months on study at the time of the protocol amendment that allowed additional treatment during months 12–24 months. Of the 14 that were eligible, 13 were offered additional treatment. In the 18 patients with complete VEGF-D data, percent change in tumor size and VEGF-D levels at 12 months is similar between the two groups (see Tables S1 and S2). Although baseline tumor size was similar in the group with missing 24 month VEGF-D data (n = 10, see Table S3), there was a slightly higher percent decrease in tumor size in that group at 12 months. The impact of the response difference in this group on our 24 month VEGF-D analysis reported here is unknown.

VEGF-D has been investigated previously primarily in patients with LAM but the underlying mechanism leading to elevated VEGF-D in patients with angiomyolipomas and pulmonary LAM remains unknown. Seyama and colleagues were the first to report elevated VEGF-D levels in a population of 44 women with sporadic LAM [3]. They also reported a negative correlation between VEGF-D levels and FEV1/FVC. In a study of VEGF-D levels in 111 women with sporadic LAM and 40 healthy controls [2], elevated VEGF-D levels were observed primarily in women with LAM who also had lymphatic involvement, regardless of whether kidney angiomyolipomas were present. They also reported a correlation between VEGF-D levels and CT scan grade for severity of lung disease. Using a grading scale of I (milder disease with cysts involving less than one third of the lung parenchyma) to III (more severe disease with cysts involving more than two thirds of lung parenchyma), they found that VEGF-D levels were higher in women with CT scan grades of II and III compared with those with CT scan grade I (p = 0.033). In 2008, Young and colleagues reported the potential diagnostic utility of VEGF-D levels for women with cystic lung disease of unknown etiology as elevated VEGF-D levels were associated with LAM [4], but not healthy women or women with other lung diseases. In a follow-up prospective study, this group reported that in a cohort of 48 women presenting with cystic lung disease, VEGF-D levels of >800 pg/ml were diagnostically specific for LAM, whereas levels <600 ng/ml were associated with other causes of cystic lung disease [6]. In a phase 3 multicenter trial (MILES Trial) evaluating the efficacy of sirolimus for the treatment of LAM (sporadic and TSC associated), VEGF-D levels were elevated at baseline (2029±2343 pg/ml, all subjects, n = 89) and decreased in the group treated with sirolimus for 12 months (862±540 pg/ml, n = 41), but not in the placebo group (2444±3862 pg/ml, n = 34). The change from baseline for the sirolimus versus the placebo group was significantly different (p = 0.001). A major difference in our study population was that we recruited subjects with kidney angiomyolipomas. Consequently, not all of our subjects had LAM, and those with LAM were likely to have less severe pulmonary disease than in the LAM cohorts used for the VEGF-D studies described above. We did observe higher levels of VEGF-D in TSC/LAM patients (compared with TSC only patients) and women (versus men), which is similar to findings reported by Young and colleagues (2008).

Although it is clear that VEGF-D is an interesting biomarker, the mechanism leading to elevated VEGF-D in patients with angiomyolipomas and pulmonary LAM is not currently known. Furthermore, the role of elevated VEGF-D in the development and/or progression of disease is also not understood. It is known that VEGF-D binds the receptor VEGF-R3 and this interaction is important for lymphangiogenesis [1]. There is some evidence that the interaction between VEGF-D and VEGF-R3 on pulmonary LAM cells may induce proliferation of these abnormal cells in the lung. One group of investigators has shown that VEGF-D stimulates migration and proliferation of LAM derived cells. They also used immunochemistry to show that VEGF-R3 is present on LAM derived cells [25]. There is some debate about these findings as another group has isolated LAM cell clusters and lymphatic endothelial cells from the chylous effusions of LAM patients and found that VEGF-R3 expression is present on lymphatic endothelial cells but not on LAM cell clusters [26]. There have also been reports of solid tumors that express VEGF-R3. However, the reliability of some of these data has been questioned because several antibodies that were used may have been non-specific. Based on an extensive review of the literature and detailed analysis (including northern, FACS, western blotting, RT-PCR, immunoprecipitation and immunohistochemistry) of 62 tumor cell lines and staining of 456 tumor tissues (including 35 histological types), one group reported that expression of VEGF-R3 is negligible in most solid tumor cells when compared to vascular and lymphatic endothelial cells. They concluded that VEGF-R3 expression is restricted to endothelial and lymphatic cells or tumors of blood/lymph origin [27]. Consistent with this conclusion, in a study of 28 malignant melanoma tumor samples, VEGF-D expression was observed in the melanoma tumor cells and both VEGF-D and VEGF-R3 were expressed on nearby endothelial cells [28]. Achen and colleagues (2001) also note that this pattern is consistent with a paracrine mechanism in which tumor cells secrete VEGF-D and VEGF-D binds VEGF-R3 expressed on nearby endothelial cells which then internalize the VEGF-D. Interestingly, it has also been reported that elevated VEGF-D in kidney confined renal cell carcinoma was associated with improved disease survival [29]. There are some intriguing observations regarding VEGF-D and VEGF-R3 expression in solid tumors and LAM cells that may be relevant to our findings in patients with kidney angiomyolipomas, however no definitive mechanism has been confirmed. As there currently are no data available on the expression of VEGF-D or VEGF-R3 in angiomylipomoma tissues or cells, additional studies are needed to understand the importance of VEGF-D/VEGF-R3 interactions relevant to the development and progression of this prominent feature of tuberous sclerosis and sporadic LAM.

In conclusion, our results show that serum VEGF-D may be useful for monitoring response to treatment with sirolimus and kidney angiomyolipoma size. Because of the study limitations, confirmation of these findings in future randomized studies with larger numbers of TSC patients and longer duration follow-up are needed before this could be recommended for use in a clinical setting. Since TSC is usually diagnosed during childhood and kidney angiomyolipomas are common in children greater than five years of age, future studies that include pediatric subjects would be of clinical importance because there may be the potential to identify individuals at risk for developing problematic kidney angiomyolipomas early in the disease process. If VEGF-D does prove to be useful for monitoring the severity of these tumors, it may allow adequate monitoring with less frequent kidney MRI or other imaging tests. A clear understanding of the mechanism of VEGF-D signaling in the development and progression of kidney angiomyolipomas associated with TSC will require further research.

## Supporting Information

Figure S1 **Percent change in kidney angiomyolipomas size at 12 and 24 months for individual subjects.** A) Subjects from the ON SIROLIMUS AFTER 12 MONTHS subgroup (n = 6); B) Subjects from the OFF SIROLIMUS AFTER 12 MONTHS subgroup (n = 12). The blue bars show the percent change in kidney tumor size at 12 months. The red bars show year 2 measurements of kidney tumors in treatment subsets (24 months). Arrows indicate return to baseline (0% change). Note: the subject with the tumor size increase of ∼60% had a single small tumor at baseline. In this case, the tumor diameter was 2.2 cm at baseline, 3.2 cm at 4 months, 2.9 cm at 8 months, 1.8 cm at 12 months, 3.5 cm at 18 months, and 3.5 cm at 24 months. Because of the small size of the tumor, we suspect that the changes noted during our study may be in part due to imaging slightly different tumor cross sections at different time points. All details regarding timing of response, number of tumors per subject, and kidney tumor size have been published in reference 19 (Table S1).
(DOC)

Figure S2 **Serum levels of VEGF-D at baseline and 12 months of sirolimus treatment in patient subgroups.** VEGF-D levels decrease in all subgroups (males, females, TSC only, TSC/LAM) at 12 months. As indicated, the decrease is statistically significant (using both Mann-Whitney test and paired t test) in females and subjects with TSC/LAM, but not in males or patients with TSC only. The horizontal lines are geometric means. Upper p values were determined using the Mann-Whitney test and lower p values (in italics) were determined using the t test (paired t test for comparing baseline to 12 months; unpaired t test to compare other indicated subgroups).
(PPT)

Figure S3 **VEGF-D levels do not correlate with pulmonary function testing at baseline.** Graphs of indicated baseline pulmonary function test and corresponding serum VEGF-D levels for individual subjects with TSC/LAM. There was no evidence for a correlation between VEGF-D levels and FVC (A), FEV1 (B), DLCO (C), or FEV1/FVC (D).
(DOC)

Figure S4 **VEGF-D levels and SEGAs.** The mean baseline VEGF-D level was higher in subjects with SEGAs (TSC related brain tumor) compared to those without SEGAs, however the difference was not significant (p = ns). Horizontal lines are geometric means.
(DOC)

Table S1 **Kidney tumor sizes for both subgroups at baseline, 12 months, 24 months.**
(XLS)

Table S2 **VEGF-D levels for both subgroups at baseline, 12 months, 24 months.**
(XLS)

Table S3 **Kidney tumor size at baseline and 12 months for subjects with missing VEGF-D data at 24 months.**
(XLS)
