# Supplementary figures and images for: Similar Trends in Serum VEGF-D Levels and Kidney Angiomyolipoma Responses with Longer Duration Sirolimus Treatment in Adults with Tuberous Sclerosis
Source: PLoS One. 2013 Feb 20;8(2):e56199. doi: 10.1371/journal.pone.0056199 (PMC3577773; doi:10.1371/journal.pone.0056199)

## Slide 1
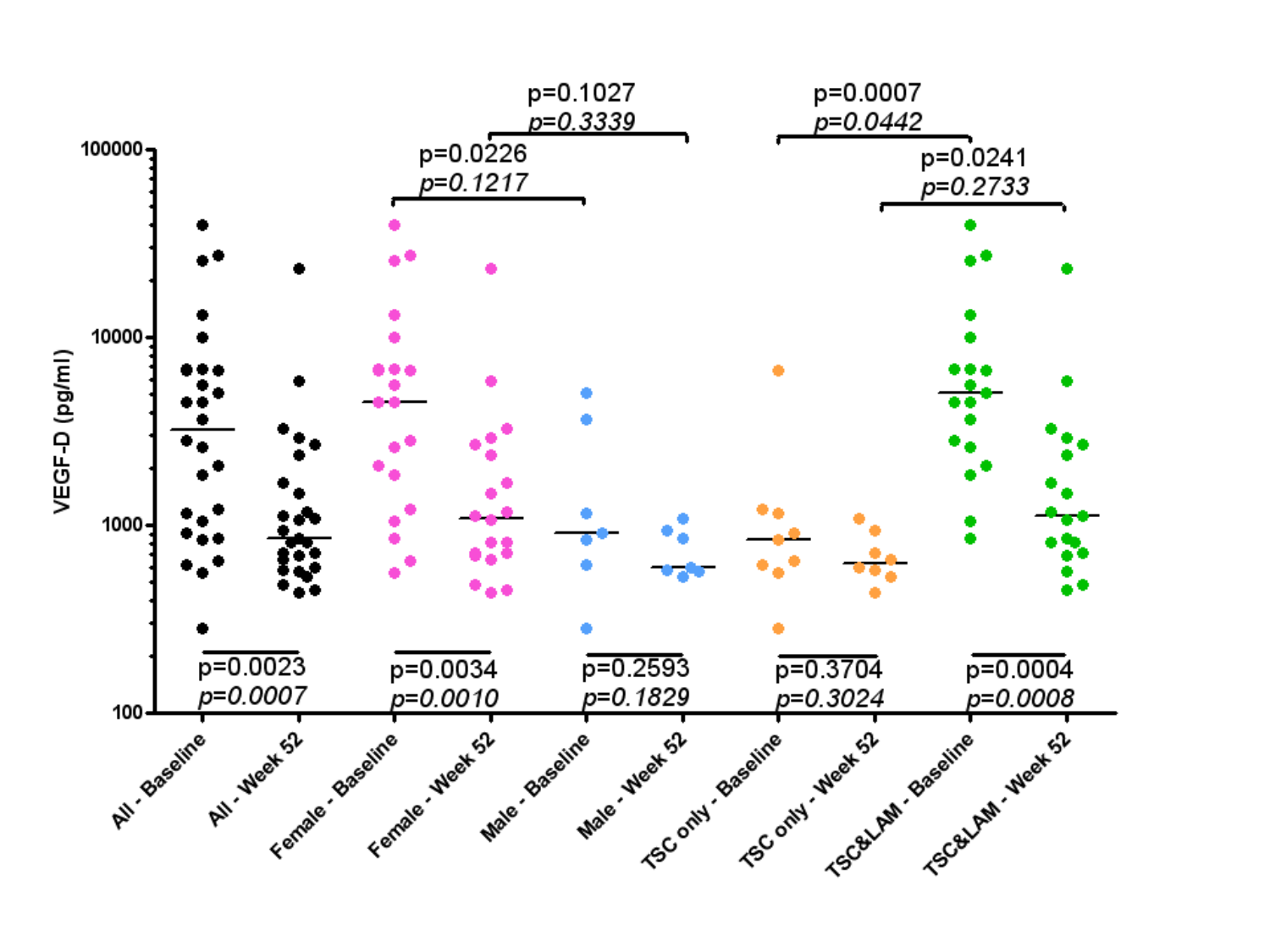

Supplement: Figure S2 — Serum levels of VEGF-D at baseline and 12 months of sirolimus treatment in patient subgroups. VEGF-D levels decrease in all subgroups (males, females, TSC only, TSC/LAM) at 12 months. As indicated, the decrease is statistically significant (using both Mann-Whitney test and paired t test) in females and subjects with TSC/LAM, but not in males or patients with TSC only. The horizontal lines are geometric means. Upper p values were determined using the Mann-Whitney test and lower p values (in italics) were determined using the t test (paired t test for comparing baseline to 12 months; unpaired t test to compare other indicated subgroups). (PPT) [file pone.0056199.s002.ppt]
