# Supplementary material for: Similar Trends in Serum VEGF-D Levels and Kidney Angiomyolipoma Responses with Longer Duration Sirolimus Treatment in Adults with Tuberous Sclerosis
Source: PLoS One. 2013 Feb 20;8(2):e56199. doi: 10.1371/journal.pone.0056199 (PMC3577773; doi:10.1371/journal.pone.0056199)
Supplement: Supplementary file 2 [file pone.0056199.s001.doc]

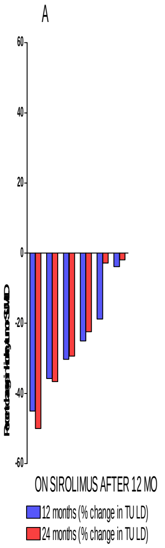

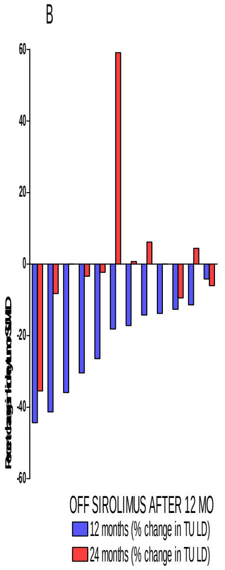

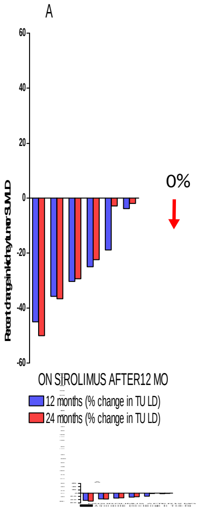

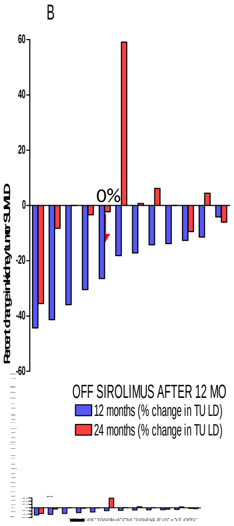

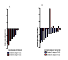
12 months (% change in TU SUM LD)
24 months (% change in TU SUM LD)

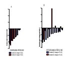
12 months (% change in TU SUM LD)
24 months (% change in TU SUM LD)
