# Supplementary material for: Similar Trends in Serum VEGF-D Levels and Kidney Angiomyolipoma Responses with Longer Duration Sirolimus Treatment in Adults with Tuberous Sclerosis
Source: PLoS One. 2013 Feb 20;8(2):e56199. doi: 10.1371/journal.pone.0056199 (PMC3577773; doi:10.1371/journal.pone.0056199)
Supplement: Supplementary file 3 [file pone.0056199.s003.doc]

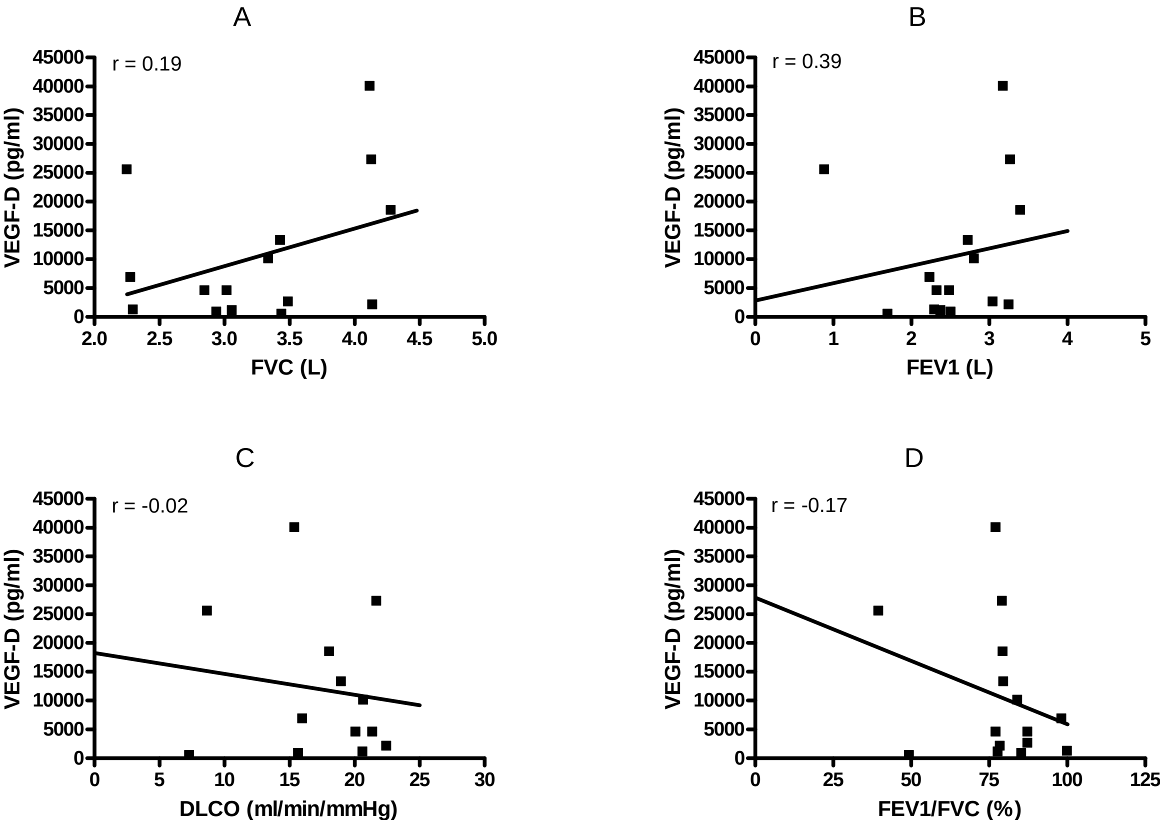
A
r = 0.19
VEGF-D (pg/ml)
45000
40000
35000
30000
25000
20000
15000
10000
5000
0
2.0 2.5 3.0 3.5 4.0 4.5 5.0
FVC (L)
B
r = 0.39
VEGF-D (pg/ml)
0 1 2 3 4 5
FEV1 (L)
C
r = -0.02
VEGF-D (pg/ml)
0 5 10 15 20 25 30
DLCO (ml/min/mmHg)
D
r = -0.17
VEGF-D (pg/ml)
0 25 50 75 100 125
FEV1/FVC (%)
