# Supplementary material for: Similar Trends in Serum VEGF-D Levels and Kidney Angiomyolipoma Responses with Longer Duration Sirolimus Treatment in Adults with Tuberous Sclerosis
Source: PLoS One. 2013 Feb 20;8(2):e56199. doi: 10.1371/journal.pone.0056199 (PMC3577773; doi:10.1371/journal.pone.0056199)
Supplement: Supplementary file 4 [file pone.0056199.s004.doc]

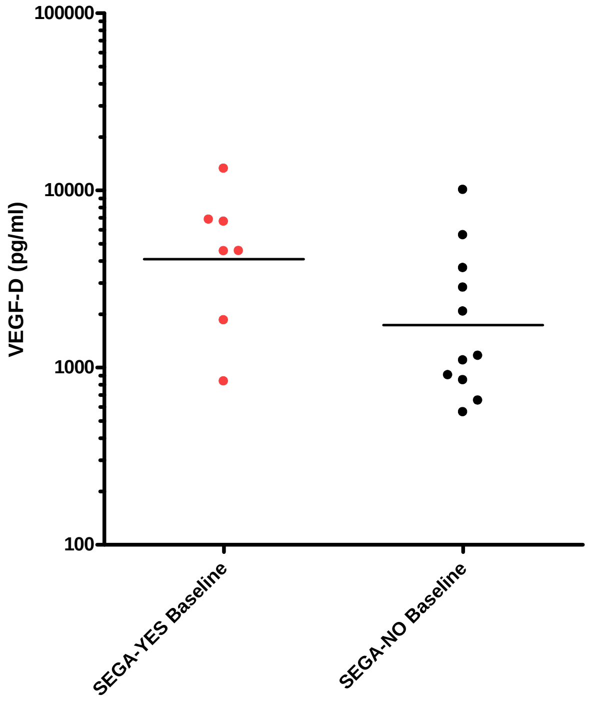
100000
10000
1000
100
VEGF-D (pg/ml)
SEGA-YES Baseline
SEGA-NO Baseline
